# Dimensions of sustainability for a health communication intervention in African American churches: a multi-methods study

**DOI:** 10.1186/s13012-017-0576-x

**Published:** 2017-03-28

**Authors:** Mary Ann Scheirer, Sherie Lou Z. Santos, Erin K. Tagai, Janice Bowie, Jimmie Slade, Roxanne Carter, Cheryl L. Holt

**Affiliations:** 1 Scheirer Consulting, Princeton, NJ USA; 2 Department of Behavioral and Community Health, University of Maryland, School of Public Health, College Park, MD USA; 3 Department of Health, Behavior and Society, Johns Hopkins Bloomberg School of Public Health, Baltimore, MD USA; 4 Community Ministry of Prince George’s County, Seat Pleasant, MD USA

## Abstract

**Background:**

Sustainability of evidence-based health promotion interventions has received increased research attention in recent years. This paper reports sustainability data from Project HEAL (Health through Early Awareness and Learning) a cancer communication implementation trial about early detection, based in African American churches. In this paper, we used a framework by Scheirer and Dearing (Am J Publ Health 101:2059-2067, 2011) to evaluate multiple dimensions of sustainability from Project HEAL.

**Methods:**

We examined the following dimensions of sustainability: (a) continued benefits for intervention recipients, (b) continuation of intervention activities, c) maintaining community partnerships, (d) changes in organizational policies or structures, (e) sustained attention to the underlying issues, (f) diffusion to additional sites, or even (g) unplanned consequences of the intervention. Project HEAL provided a three-workshop cancer educational series delivered by trained lay peer community health advisors (CHAs) in their churches. Multiple sources of sustainability were collected at 12 and 24 months after the intervention that reflect several levels of analysis: participant surveys; interviews with CHAs; records from the project’s management database; and open-ended comments from CHAs, staff, and community partners.

**Results:**

Outcomes differ for each dimension of sustainability. For *continued benefit*, 39 and 37% of the initial 375 church members attended the 12- and 24-month follow-up workshops, respectively. Most participants reported sharing the information from Project HEAL with family or friends (92% at 12 months; 87% at 24 months). For *continuation of intervention activities*, some CHAs reported that the churches held at least one additional cancer educational workshop (33% at 12 months; 24% at 24 months), but many more CHAs reported subsequent health activities in their churches (71% at 12 months; 52% at 24 months). No church replicated the original series of three workshops. Additional data confirm the *maintenance of community partnerships*, some changes in church health *policies*, and *continued attention to health issues* by churches and CHAs.

**Conclusions:**

The multiple dimensions of sustainability require different data sources and levels of analysis and show varied sustainability outcomes in this project. The findings reinforce the dynamic nature of evidence-based health interventions in community contexts.

**Electronic supplementary material:**

The online version of this article (doi:10.1186/s13012-017-0576-x) contains supplementary material, which is available to authorized users.

## Background

An essential long-term outcome of dissemination and implementation research is sustaining the target intervention within its setting [1–3]. Sustainability can be defined as “the continued use of program components and activities for the continued achievement of desirable program and population outcomes” [4]. Other terms used by prior researchers include continuation, confirmation, maintenance, durability, continuance, and institutionalization. Sustainability has received increased research attention in recent years [3, 5–7], but data-based literature illustrating multiple types of sustainment outcomes is nearly non-existent. This paper reports sustainability data from Project HEAL (Health through Early Awareness and Learning) a cancer communication implementation trial about the need for early detection, based in African American churches. We report data from multiple measures of sustainability to illustrate the multiple levels of analysis necessary to fully assess the sustainability of health promotion interventions.

Interest and research about the long-term sustainability of health-related interventions is growing but often has not operationalized the term “sustainability” as a set of multiple outcomes [3, 5, 6, 8–10]. Researchers, funders, policy-makers, and community practitioners are concerned whether their interventions continue after an initial research project or grant [1, 11–13]. Recent advice for researchers also emphasizes the dynamic nature of these processes, as factors in community organizations and contexts interact over time to produce or inhibit long-term sustained program outcomes [7, 14].

The importance of the multiple dimensions of sustainability for long-term improvement of community outcomes should not be underestimated. Organizations delivering the interventions must be able to continue implementing program activities for community-based interventions to affect the lives of those they are intended to serve. Given the considerable health disparities that continue to affect medically underserved populations [15], it is important to sustain evidence-based interventions that are delivered in community settings in particular because many of these settings may be limited in resources. Assessing the full sustainability of interventions in underserved communities thus requires a more detailed approach to measurement and data collection than simply asking “Was the project sustained?” For example, involving a broader population requires that the innovative ideas and strategies are spread to other organizations and implementers.

Current research about sustainability has highlighted the importance of factors or processes that increase the likelihood of sustaining an intervention (predictors of sustainability) [6, 7, 16]. Other researchers discuss sustainability as a set of processes or capacities that take place during the initial implementation of a program [17, 18], but this approach does not require longer-term data about actual continued implementation or benefits for consumers. In contrast to the focus on processes to increase sustainability, a more detailed conceptual framework for research about sustainability proposes that multiple types of long-term outcomes should be differentiated when reporting research about sustainability [4]. Few if any publications detail empirical results for diverse sustainability outcomes using multiple measures from an intervention project. This paper illustrates empirical examples of multiple measures of sustainability for a cancer communication intervention in African American churches.

Scheirer and Dearing [4] recognized the multi-dimensional nature of the concept of sustainability and proposed that six types of outcome measures should be differentiated. This typology of sustainability outcomes was derived from its authors’ prior research and experience, as well as from discussions in two large-scale workshops involving numerous researchers about sustainability measures. These types of sustainability outcome measures can be summarized as follows:

- Whether *benefits or outcomes for consumers/clients/participants* are continued (when the intervention provides services to individuals).
- Continuing the *program activities or components* of the original intervention within the same or similar organizations, especially by specifying the active components of the interventions and assessing the extent that each is modified during a follow-up period.
- *Maintaining community-level partnerships or coalitions* developed during the funded program, with a potential for additional or supplementary activities along with enhanced community capacity.
- *Maintaining new organizational practices*, *procedures*, *and policies* that were started during program implementation, which may contribute to continued implementation of the target program or to starting up new activities.
- *Sustaining attention* to the issue or problem that can lead to increased public awareness and ultimately greater resources from organizational or political sources.
- *Program diffusion and replication* into other sites where the underlying concepts or specific activities spread to use in other organizations, with the extent of dissemination activity by the initial implementers as a proxy indicator for the extent of diffusion that might occur.

These diverse types of sustainability outcomes require different levels of analysis (e.g., data collected from individual participants in the program, data to assess the continuation of the program activities, broader considerations of organizational or community-level change). It is not possible to address all components of sustainability with any one method of data collection and analysis. This adds complexity and cost to attempts to measure the extent of sustainability for a specific project because data about any one level of sustainability outcome does not necessarily encompass other levels of these outcomes. These complexities are illustrated in this multi-method study of Project HEAL sustainability in African American churches. We were interested not only in whether the intervention was continued as originally implemented but also if it was modified or unfolded differently over 24 months.

## Methods

### Project HEAL—the intervention and research design

African American churches have been identified as an important venue for addressing inequities in cancer and other health conditions [19–24]. Project HEAL compared two approaches to training lay peer community health advisors (CHAs) to implement an evidence-based cancer educational curriculum in African American churches: a “Traditional” versus a web-based “Technology” approach. The 15 participating African American churches were randomly assigned to either the traditional or the web-based training approach. Each church recruited one male and one female to receive CHA training and certification, who then led a series of three cancer education workshops for their church members (breast, prostate, and colorectal; see Holt et al. [25] for a full description of the intervention). Results for implementation and efficacy outcomes are reported in other papers (Holt CL, Tagai EK, Santos SLZ, Scheirer MA, Bowie J, Haider M, Slade J, Wang MQ, Whitehead T: Is online comparable to in-person training for community health advisors conducting a church-based intervention? 2017. Forthcoming.) [26]. For the overall study, 15 churches were recruited and randomized, 30 CHAs trained, and 42 workshops conducted, serving 375 member participants from those churches

The “Traditional” CHA training approach used in-person classroom training for the CHAs to learn about breast, prostate, and colorectal cancer early detection. CHAs then recruited participants from members of their churches to attend the series of workshops. These workshops provided evidence-based intervention content based on the prior research of the principal investigator for this implementation and sustainability research project [21, 27, 28]. The content of CHA training and intervention workshops in the “Technology” approach were the same, except the CHAs completed their training using a web-based system and with less technical assistance than was provided to the traditionally trained CHAs [25, 29]. Technical assistance could range from providing assistance maneuvering through the web-based training system to answering questions about the content of the training and the workshop format. CHAs from both types of training were then responsible for presenting the three in-person workshops for participant members recruited from their churches. The intervention workshop series was to be held within 2 months of completing CHA training, and the CHAs were encouraged, but not required, to later replicate one or more of the workshops.

For both study groups, follow-up workshops were held at each church about 12 and 24 months after the intervention workshop series to collect survey data from participants and CHAs, provide recap sessions about cancer screening, and report back data from the baseline survey. Additional engagement was maintained with CHAs and participants through newsletters (sent quarterly after completion of the workshop series, throughout the 24-month study period), mobile phone text messages (sent every other week to CHAs and participants who opted to receive them, throughout the 24-month study period), annual holiday events each December, in-person interviews with pastors shortly after the completion of the workshop series, and interviews with CHAs at 12- and 24-months to encourage their continued involvement. Sustainability of health outcomes reported in this paper were collected from participants and CHAs during the 12- and 24-month workshops (Table 2).

Project HEAL is a capacity building intervention. By training CHAs as health educators in the churches starting with a focus on cancer early detection, we recognized that there was community interest in additional areas of chronic disease beyond cancer (e.g., heart disease, diabetes), and encouraged and anticipated that the CHAs would naturally expand their educational activities to these areas of interest once the initial Project HEAL three-workshop cancer series was completed. While not required, we believe that this is a strength of the CHA intervention approach and that it would foster this expression of sustainability. Project staff did not do anything specifically to foster sustainability other than encourage the CHAs to continue their health promotion activities and provide technical assistance to them upon request. Study staff did ask the CHAs and study participants about their interest in additional health activities at the 12-month follow-up workshop.

Churches, CHAs, and participants received monetary incentives as well as articles of appreciation with the HEAL logo, such as tote bags, bookmarks, pens, and a recognition plaque for each church. Community involvement, especially recruitment of churches, was facilitated by input from several meetings of a community advisory group and by a longstanding partnership with Community Ministry of Prince George’s County (Community Ministry), a 41-year-old non-profit organization providing convening and collaborative activities among faith-based organizations.

#### CHA demographics

CHAs tended to be active volunteer leaders in their churches (two CHAs were also the pastors) and were ages 25 to over 70 (mean age of 51). About two-thirds cited a bachelor’s degree or higher as their educational level. Seventeen (61%) of the initial 28 CHAs who provided background data were currently employed, 7 of them in health-related professions or jobs (another 6 were retired, 1 on disability, and 4 did not state their employment status). Facilitating cancer awareness education may have been personally relevant to many CHAs, as 16 of the initial 28 (57%) had experienced cancer, either themselves or through family members.

#### Workshop participant demographics

Individuals who enrolled in Project HEAL (*N* = 375) were mostly female (68%) with an average age of 55.28 (SD = 9.28) (Holt CL, Tagai EK, Santos SLZ, Scheirer MA, Bowie J, Haider M, Slade JL: Web-based vs. in-person methods for training lay community health advisors to implement health promotion workshops: Participant outcomes from Project HEAL. 2016. Forthcoming.) [26]. These participants were middle class with a median education including some college and a median family income in the $50–60,000/year bracket. Almost half (47.68%) were married and more than half reported working full-time (53.53%). Most (93.07%) had health insurance coverage.

#### Overall project implementation

In general terms, implementation was successful: although two churches dropped out early in the project and one was replaced (the second church could not be replaced due to project timeline), 14 of the 15 initial churches continued through the initial intervention phase and 93% of the intended workshops for all 15 churches were facilitated by the CHAs [26]. All 14 churches held the follow-up sessions for participants about 12 and 24 months after their first workshop, which were facilitated by the research staff for both training conditions.

### Data collection methods for Project HEAL sustainability research

The RE-AIM (Reach Effectiveness Adoption Implementation Maintenance) Framework [30] was used to assess implementation outcomes such as adoption, reach, implementation, efficacy (reported elsewhere (Holt CL, Tagai EK, Santos SLZ, Scheirer MA, Bowie J, Haider M, Slade JL: Web-based vs. in-person methods for training lay community health advisors to implement health promotion workshops: Participant outcomes from Project HEAL. 2016. Forthcoming.) [26]), and sustainability (maintenance) outcomes across a 24-month follow-up period. During the initial series of workshops, data were collected via participant surveys (see Additional files 1, 2, 3, and 4), observations by project staff, implementation checklists at workshops, and interviews with the CHAs (see Additional files 5 and 6) and church pastors. Some missing data at each level resulted in variations in available sample sizes for several measures. In cases where 24-month data is not presented, these indicators were not collected.

Table 1 summarizes the dimensions of sustainability, data sources, and example indicators and indicates when the data was collected. Multiple methods were used to collect data to assess sustainability approximately 12 and 24 months following the start of the workshop series in each church. Due to the unavailability of an appropriate standardized sustainability tool, the study team met early in the development of Project HEAL, inventoried all study measures for indicators of sustainability and added some items as needed, using the Scheirer and Dearing [4] framework as a stimulus. These multiple data sources allowed us to assess several conceptual definitions of project sustainability suggested by the Scheirer and Dearing [4] framework.

**Table 1:** Sustainability outcomes illustrated by project HEAL

Sustainability outcome	Project HEAL data source	Time of data collection	Example indicators
a. Continued benefits for consumers/clients/participants	Workshop attendance	12 and 24 months	• # of participants attending 12- and 24-month workshops
	Participant surveys^a^	12 and 24 months	• # of participants sharing information from Project HEAL with others
	CHA interviews^b^	12- and 24-months	• # of church members participating in additional workshops
b. Continued Project HEAL workshops	CHA interviews	12 and 24 months	• # of additional cancer workshops
Other health promotion activities	CHA interviews	12 and 24 months	• # of additional health activities
c. Maintaining community-level partnerships or coalitions	Staff and community partner records	Ongoing throughout project	• Subsequent joint research/grant applications and funding
	CHA interviews	12 and 24 months	• # of collaborative health activities (e.g., health fair)
d. Maintaining new organizational practices, procedures, or policies	CHA interviews	12 and 24 months	• Health ministry development/planning
			• Church health policy development/modification
b. Sustaining attention to the issue or problem	CHA interviews	12 and 24 months	• Subsequent health activities/policies
			• CHA continuing health education^c^
f. Program diffusion and replication to other sites	CHA interviews	12 and 24 months	• # of Project HEAL churches contacted by other churches about health^d^
	Staff records	Ongoing throughout project	

Scheirer MA, Dearing JW: An agenda for research on the sustainability of public health programs. *American Journal of Public Health* 2011, 101:2059-2067.

^a^See Additional files 1, 2, 3, and 4

^b^See Additional files 5 and 6

^c^Note assessed at CHA 24-month interview

^d^Data obtained from both CHA interviews and staff records

Paper surveys were completed by church member participants in Project HEAL at the 12- and 24-month follow-up workshops. Participants that did not attend the follow-up workshops were mailed the survey, followed by phone calls and emails. A shortened version was sent to initial non-respondents. We obtained responses from 298 of the 375 participants, a 79% participant response rate at 12 to 18 months following the intervention workshops. Three hundred nine participants completed the participant survey approximately 24 months post-workshop series resulting in an 84% response rate (*N* = 366).

Semi-structured interviews with 24 CHAs (80% of the 30 originally trained CHAs) were conducted by Project HEAL staff either in-person or by telephone, 12 and 24 months following the initial workshop series. These interviews were recorded by the interviewer using a structured response option format, with a few open-ended items. The interviewers had been trained previously on interviewing techniques, when they also interviewed church pastors during the first year of the project.

Quantitative data analysis was conducted using SPSS and largely focused on descriptive analyses such as frequency distributions. Analysis of the few open-ended questions took a content analysis [31] approach where it was reviewed for common themes (without an a priori codebook). Data were analyzed for any differences by study group in CHA training methods; however, there were no differences and so for parsimony we present data collapsed across study groups. Project staff maintained a tracking database of participant attendance and staff contacts with each church and CHAs over time, which included church characteristics. The community partner and Project HEAL staff provided descriptive examples of other activities related to Project HEAL in the churches, the community, and their own activities.

## Results

### Results for multiple dimensions of sustainability

The data collected from these multiple methods illustrate several perspectives about the sustainability of Project HEAL approximately 12 and 24 months after the start-up of its intervention workshops. The results presented below follow the framework of potential sustainability outcomes suggested by Scheirer and Dearing [4].

A. *Outcomes for participants* (individual-level sustainability). We assessed several sustained outcomes for the church member participants enrolled in the Project HEAL workshops, including whether those participants continued to engage in the program by attending the 12-month follow-up workshops at their churches. Our attendance records show that 39% (149) of the original 379 participants attended a 12-month follow-up workshop and 37% attended the 24-month workshop. In addition, many participants shared cancer education messages with others, which may be an important social influence to spread cancer awareness messages to others. In their responses to the 12-month survey, 92% reported that they had “shared knowledge from Project HEAL workshops” with someone else while 87% indicated doing so at 24 months. In addition, CHAs estimated that about 235 church members participated in new workshops conducted in their churches after Project HEAL (described below). We were not able to collect any outcome data from these new workshop participants.
B. *Sustained project activities*: *workshops* (CHA and church-level sustainability). Data at the CHA level are presented in Table 2. Of the 24 CHAs interviewed at 12 months, eight (33%) reported that additional cancer-related workshops were conducted in their churches, after the initial three-workshop series for Project HEAL, while 5 (24%) reported doing so at 24 months. None of the CHAs reported that they replicated the original series of three Project HEAL workshops.

**Table 2:** CHA-reported sustained health activities in the churches

	12 months	24 months
	(*N* = 24)	(*N* = 21)
	*n* (%)	*n* (%)
Replicated Project HEAL three-workshop series	0 (0%)	0 (0%)
Church conducted additional cancer workshops	8 (33%)	5 (24%)
Other health-related activities conducted in church	17 (71%)	11 (52%)
New health classes/group sessions	6 (25%)	4 (19%)
Brochures or materials distributed	13 (54%)	10 (48%)
Participated or organized health fair	10 (42%)	6 (29%)
Walking or exercise groups	5 (21%)	5 (24%)
Health promotion activities for children	4 (17%)	5 (24%)
Screening or health testing	7 29%	2 (10%)
Other health topics addressed in the churches		
Physical activity or walking	18 (67%)	10 (48%)
Healthy diet	15 (63%)	4 (19%)
Obesity or weight loss	8 (33%)	10 (48%)
Aging	5 (21%)	2 (10%)
Smoking	3 (12%)	6 (29%)
Cancer	13 (54%)	14 (67%)
Heart disease or high blood pressure	11 (46%)	8 (38%)
Diabetes	8 (33%)	7 (33%)
Stroke	5 (21%)	4 (19%)
Asthma or children’s health	3 (12%)	1 (5%)
HIV/AIDS	4 (16%)	4 (19%)
Other	9 (37%)	6 (29%)
*Other health-related activities* (church-level sustainability). Seventeen of the 24 CHAs (71% percent) reported at 12 months that additional health-related activities had been sponsored by their churches since the initial Project HEAL intervention and at 24 months this number had reduced to 52%. Eight CHAs were involved in conducting these other activities, in addition to 16 other church leaders and some outside agencies. Of the 17 CHAs that reported additional health activities in their churches at 12 months, 6 (35%) indicated that these activities were stimulated by Project HEAL. The CHAs estimated that nearly 500 church and community members had participated in these additional activities.
  Unfortunately, we cannot precisely report the extent of expansion of these other health activities as we did not explicitly record at the start of the project what health-related activities were already being conducted. It is very likely, however, that Project HEAL contributed to the churches’ capacity to provide health-related programs and activities, when church leaders became more aware of the importance of physical health in addition to the spiritual health of their members. Several CHAs commented in their 12 month interviews about the roles of the HEAL project in stimulating other health activities:
*“HEAL was an awareness alert, a wake- up call informing people.” – CHA 1*
*“The CHA spearhead it - she wanted to reach more people about health issues. Since Project HEAL, the Pastor also talks about health issues – [there are] ongoing health messages from our Pastor.” - CHA 2*
*“We had previously done a health fair, but Project HEAL opened up the health fair to more topics (especially cancer) and a grander scale. It was a bigger health fair in summer 2014. Attendance was large - it was opened up to community members as well.” - CHA 3*
C. *Community partnerships maintained* (partnership-level sustainability). The Project HEAL staff continued their contacts with participating churches by organizing the follow-up workshops, sending out quarterly newsletters to participants and CHAs, distributing text messages to member participants, and inviting pastors and CHAs to annual holiday gatherings. Successful partnership with Community Ministry is continuing, as evidenced by the joint preparation of several additional grant applications, subsequent grant support, and reciprocal participations in each other’s activities. Several participating churches have since contacted the Community Ministry to request and receive technical assistance, for example, in conducting additional outreach events concerning cancer or about health insurance. Staff members from the University of Maryland and its partner organization frequently participate in health fairs sponsored by Project HEAL churches and other community organizations.
  When describing other health-related activities that their churches had sponsored since Project HEAL, several CHAs mentioned additional organizations they had partnered with in those activities, including a health department, a Christian radio station, community members who were not members of their own church, and the organizers of a state health fair. We note that Project HEAL was not specifically intended to develop relationships with community partners, but these relationships were continuously fostered among churches and with the university when developing other health-related activities.
D. *New health policies or procedures by churches* (church-level sustainability). A deeper form of sustainability occurs when an organization makes permanent changes in its structures or policies, for example, to form health ministries, to initiate wellness policies, or to obtain other resources for health programs. The community partner reported that several churches have health ministries with multiple ongoing activities. Several churches started web pages or newsletters that include monthly updated health promotion topics. For example, one church’s website lists a Health Awareness Ministry to focus on improving health, with a new health topic introduced monthly. In the data from the 12-month CHA interviews, five CHAs (21%) reported their church had formed a health ministry or health committee since Project HEAL began (14% at 24 months), and six others were interested in establishing one. Twenty-one CHAs (88%) provided narrative information about their suggested plans for their health ministries at 12 months, with descriptions of ideas such as the following:
*“More classes frequently and [more] topics. Want to cover all topics in the health topic list. Need to find time in church calendar, perhaps one time per month - may not be able to hold weekly.” - CHA 4*
*“Our will is to continue with the Project HEAL project this year and our community health fair with [Church name]. Continue with our exercise group and to make children cancer aware”. - CHA 5*
*“To continue empowering the people with this knowledge.” - CHA 6*
  Few churches had formal wellness policies at the time of the 12-month follow-up interviews: only one CHA reported having a formal church wellness policy (two more at 24 months), but seven others were not sure if there was a policy (three were not sure at 24 months). However, 11 CHAs (46%) reported at 12 months that there had been changes in church policies involving health. All of these CHAs described serving healthier foods at church (although for several, this was not viewed as a *change* in policy) and eight reported their church had “no smoking” policies. The community partner confirmed that several participating churches have started serving only nutritious food at church, to teach congregants the value of preparing and serving healthier food.
E. *Sustained attention to health issues* (CHA or church-level sustainability). The information detailed above about additional activities and changes in church policies provides evidence of sustained attention to health-related issues in these churches. The diverse types of new health-related activities in churches, including health ministries, new activities, newsletters, and one with a website, will help sustain their members’ attention about these issues. In addition, many of the individual CHAs continued learning about health topics 12 months after their training for Project HEAL: 13 of the 24 (54%) had engaged in continued education about cancer, while 16 (67%) had learned about other health topics. For a few, this continuing education was part of keeping up with their requirements for employment, while at least nine obtained health information from the internet for their personal interest. They commented:
*“I joined two other organizations (Komen and DC Divas and Pink) to get involved for (1) information for the church and (2) to become an advocate.” - CHA 7*
*“I do personal research on cancer and how to tackle it. Focus on healthy lifestyle and alternative treatments (teas, nutrition, decrease sugar (alkaline environment))…In process of researching cancer, I follow up on other topics (e.g., healthy diet/nutrition, diabetes, hypertension, scientific processes).” - CHA 8*
  A pastor interviewed for the Project HEAL newsletter explained how the project has affected his church: “*Greatly, because a lot of individuals were pretty much doing nothing in terms of getting checked. Everybody really embraced it…..It’s going to change the way we deal with health for a good while.”*
F. *Diffusion to other sites* (community-level sustainability). Broader level sustainability of materials and information from Project HEAL could occur if other churches learn about it from the Project HEAL CHAs or churches. At 12 months, five CHAs (21%) replied “yes” to a question about whether any other organizations or individuals contacted their church for information about Project HEAL or other health activities, while nine others were not sure if their church had been contacted. These contacts included two other churches, as well as via “*denomination-level presentations at conferences*.” Further, a staff member from Community Ministry reported that another church, not enrolled in Project HEAL, contacted them to request information about Project HEAL and for technical assistance for their own outreach events about cancer awareness. The university has had discussions with a Christian radio station for potentially publicizing links to the Project HEAL website, where other churches can download the Project HEAL training materials.
G. *Unintended, unplanned consequences*. During the HEAL team meeting when we brainstormed potential sustainability outcomes, several community members suggested that we need to be alert for other potential consequences or outcomes. The CHA interviews asked whether there were “any outcomes from Project HEAL that were not initially planned.” None of the CHAs mentioned any unplanned negative consequences from Project HEAL. However, a Community Ministry staff member reported that they delayed working aggressively with one church requesting assistance about implementing outreach for new health insurance opportunities, to avoid conflicting with follow-up events and data collection for that church’s HEAL participants.

## Discussion

These examples about the multiple dimensions of sustainability for Project HEAL reinforce the diverse meanings of sustainability concepts and demonstrate the utility of the Scheirer and Dearing [4] framework. Overall, this evaluation of sustainability showed diverse results, depending on how sustainability was operationalized at different levels of analysis. By looking overall at the participant and CHA-level data, sustainability slightly attenuated from 12 to 24 months. However, sustainability was still fairly robust given that we relied on volunteer lay CHAs, many of whom are already over-extended in multiple volunteer roles in their churches, to conduct additional health promotion activities beyond the initial Project HEAL intervention and with no additional resources. We assessed many dimensions of sustainability at different levels of analysis: the church level, the CHA level, and the participant level. These data for Project HEAL sustainability ranged from 0% sustainability using a conservative definition—whether the churches replicated the Project HEAL three-workshop series—to 92% of participants sharing Project HEAL intervention content with other people in their lives. The CHAs reported many cancer-relevant health promotion interventions in their churches subsequent to the initial Project HEAL three-workshop series. Importantly, the CHAs also reported subsequent additional health promotion activities in other health chronic disease areas of interest to their congregations.

The Scheirer and Dearing [4] framework was useful in encouraging the team to operationalize sustainability by using not just a single measure but with multiple indicators. However, the framework did not provide standardized measures of sustainability, and there is limited guidance on interpreting the relative “weights” of the multiple indicators of sustainability that were collected. Based on the current data one could make radically different conclusions about whether Project HEAL was sustained. Perhaps a reasonable approach would be for program developers and stakeholders to identify a priori dimensions of sustainability that are most important to them at the outset of a project. Collecting data on multiple indicators of any project outcome could be burdensome for projects conducted in low-resource contexts, and some of the data can be difficult to obtain or can be subjective (e.g., sustained attention to the problem). Nevertheless, the framework provides a rich and multi-faceted way to consider the important outcome of sustainability. The Scheirer and Dearing [4] framework’s conceptualization of sustainability dovetails with that of the RE-AIM Framework [30] to the extent that both frameworks consider both individual-level and setting-level sustainability. Both consider the primary outcome and related outcomes as well as multiple indicators. The RE-AIM Framework [30] explicitly includes program adaptation, which could be more fully integrated into Scheirer and Dearing’s framework, particularly in light of the current findings that our CHAs continued many health promotion activities but not a direct replication of the Project HEAL three-workshop series.

It is likely that these diverse types of sustainability are interrelated and mutually influence the potential effects of each other. For example, the depth of involvement of the CHAs as shown by the extent of voluntary continuing education is likely to be related to their willingness to plan additional church activities, as well as their influences on the pastors for delivering health-related messages during church services. And as church members participate in some activities or hear messages from their pastor, they are likely to share their health experiences within their families and social networks, and in turn, foster requests for workshops or other information about additional health-related topics. Project HEAL provides an example of how a CHA intervention approach can build capacity in an organization and foster sustainable health promotion. The types of sustainability described as separate concepts in this paper are in reality intertwined in a potential web of factors for growing and sustaining health-related activities in these churches, beyond the specifics of the cancer awareness workshops at the heart of Project HEAL. Similar concepts about the dynamic interrelationships of evolving interventions within changing contexts have been described as the “dynamic sustainability framework” [7].

The dynamic nature of sustainability outcomes also raises questions about the contextual meaning of dissemination of “evidence-based practices” for improving health. In this case, the cancer workshops per se were based on prior research and evidence about the efficacy of similar workshops on breast, prostate, and colorectal cancer [21, 27, 28]. But the additional activities stimulated by the churches’ participation in Project HEAL may or may not have been evidence-based. For example, the CHAs reported that additional workshops about other health topics were conducted in their churches, and many churches participated in health fairs, but we have no information about the evidence base for those additional informational sources or their outcomes. The multi-faceted processes involved in this type of community participatory project means that the evidence behind their sustained delivery cannot be closely controlled. Churches and CHAs could disseminate non-evidence-based messages, as well as research-based information. This reinforces the importance of making evidence-based programs and information accessible to lay audiences and to countering the plethora of non-evidence-based messages in the popular media.

The results provided here for the diverse aspects of sustainability for Project HEAL also reinforce the complex nature of change in community contexts. In this case, there was not a linear sequence of implementing Project HEAL, then sustaining its workshops in the same or other churches. Instead, implementing Project HEAL with volunteer CHAs in diverse churches stimulated multiple streams of activities and changes in both the churches and the CHAs, which in turn are likely to foster additional types of activities among the existing networks of people. Pastors and other church leaders also became more attuned to the connections between physical and spiritual health. Throughout these activities, the roles of our community partner were crucial, from initial engagement and recruitment of the churches, to the intervention development, to the CHA training, and to implementation and evaluation. A direct “translation” of evidence-based interventions into community contexts may not be an appropriate metaphor for the dynamic processes of change that promote new health understandings and behaviors among community members.

In Project HEAL, it was initially somewhat surprising to some that the CHAs who were trained to conduct cancer educational workshops later began to conduct health promotion activities in other areas of chronic disease. We view this as a success and an indicator of sustainability in that the CHA approach is one of capacity building where CHAs are trained to impart health-related knowledge to their peers. That the CHAs started with cancer and drifted into other health areas may be viewed as a natural sustainability process, one that is supported and embraced by the Project HEAL team. However, expansion to other health topics may also be viewed as a negative unintended consequence to the extent that the CHAs reduce their focus on the original topic of interest, in this case, cancer early detection. In the end, we must balance the reality that people are whole bodies and we need to find ways to be responsive to the community’s health priorities while staying true to the primary focus of a project and yet not burning people out on a single health topic. Future research should consider thinking more purposively about sustainability and in particular, how to best support lay health educators in these efforts.

Participation in Project HEAL is likely to have increased the capacities of the churches to engage in health promotion, as CHAs learned skills and tools for conducting educational activities. In our work with churches, we view organizational capacity as a key factor in understanding outcomes along the implementation continuum, including sustainability. Churches vary greatly in their capacities, and it is likely that churches with greater baseline capacities may have had better sustainability of Project HEAL. However, there are currently limitations in capacity assessment in this type of setting that both hinder this type of analysis and provide an opportunity for future research.

These multiple types or meanings of sustainability for Project HEAL also showed diverse extents of sustainability, in measurement terms. While about half the churches sustained at least one cancer workshop, they provided larger numbers of other health-related activities. There were fewer reported examples of sustained partnerships or diffusion to other sites. Therefore, there is not a single measure of sustainability for this project that answers the question, “Was *it* sustained?” This means that predictors or influences on sustainability are also likely to differ among the different outcomes, further complicating research about factors influencing sustainability in practice. For example, to use the Program Sustainability Assessment Tool developed by Luke and colleagues [6], one would need to specify which specific sustainability outcome is being assessed, as the influencers for individual participant retention, for example, are likely to differ from influencers for church-level activity continuation or for maintenance of partnerships among organizations. In essence, it is not desirable to view sustainability as a single outcome, given the diversity of meanings and measures shown by Project HEAL.

A number of recent papers about dissemination and implementation issues have emphasized the need for better measures of the underlying constructs to promote cumulative research and to improve practice [2, 3, 32, 33]. This advice is certainly true for research about sustainability and its influences or predictors. Yet, as this paper has shown, measuring the outcomes of sustainability is not a simple task, and may yield different results depending on the unit of analysis and the data collection measures used. Although one implementation framework included “sustainability” as one outcome within a taxonomy of eight implementation outcomes [2], the data from Project HEAL has shown that sustainability is itself a multi-faceted construct. Future research about sustainability should carefully delineate and “unpack” the specific aspects of sustainability that are being measured. However, the methods for Project HEAL have demonstrated that it is feasible to collect multiple sources of data about the diverse levels of sustainability, if this important data is planned for in advance.

A priori data collection for sustainability using the Scheirer and Dearing [4] framework should certainly include multiple levels, which in the present case would be the organizational, interventionist, and participant. Continued participant benefit should consider evaluating efficacy outcomes after the intervention or the funding period has ended. Continued program activities may be better documented by talking not only with those tasked with executing those activities but also with program participants, to triangulate those perspectives on the extent to which program activities were sustained. Though we were not initially prepared to assess community-level partnerships or coalitions in Project HEAL, this information could be obtained by interviewing organizational leaders. With regard to organizational practices/procedures/policies, this information is likely best gathered from organizational leaders but again it could be triangulated or verified by asking other organizational members.

### Limitations

The current findings are limited by a number of factors. First, as with any intervention implemented in a context, specific findings may be limited in generalizability even though the broader principles are likely applicable to other interventions and contexts. Second, in the absence of baseline data about health activities that the churches or CHAs were conducting prior to Project HEAL, it is difficult to draw conclusions about whether the health promotion activities they conducted in the 24-month follow-up period were directly attributable to project HEAL. Third, Study staff did not attend and document new workshops or additional activities conducted by the CHAs subsequent to the original three workshops. Therefore, we had to rely on reports by the CHAs on these subsequent activities. Fourth, the non-standardized, context-dependent measures of sustainability are study specific and would not be applicable to other studies. These limits on the generalizability of sustainability measures are likely to be true of any research on sustainability, which needs to be intervention and context specific. This is an issue that is not unique to Project HEAL and continues to limit the field of dissemination/implementation/sustainability science.

### Implications for implementation science

The current study operationalizes and provides a data-driven illustration of the Scheirer and Dearing [4] model using Project HEAL as an example of how this framework can be used to evaluate sustainability. The current findings highlight the importance of using multiple indicators to evaluate sustainability including multiple levels, as also recommended by the RE-AIM Framework [30]. We discuss measurement challenges in evaluating sustainability and issues unique to conducting dissemination/implementation research in community settings.

## Conclusions

Research about the sustainability of community-based interventions for health promotion has increased in recent years but has not yet led to agreement on conceptual and measurement frameworks that are congruent with the complexity of the underlying processes. This paper has shown that multiple meanings and levels of analysis for sustainability exist for a single intervention project, when its potential outcomes are looked at over time. The data results for the diverse meanings of sustainability vary substantially, for example, from zero churches sustaining all three workshops with fidelity, to nearly three quarters of the CHAs reporting additional health-related activities in their churches. Future research about sustainability should specify which dimensions of sustainability it is addressing, with attention to the likely diversity of influences on those outcomes. It is not possible for research to develop policy suggestions for strengthening the extent of sustainability without first defining which dimension or meaning of sustainability is intended. For example, policies for keeping church members engaged in health promotion activities would be quite different from actions to encourage churches to adopt church wellness policies or to expand their community partnerships.

Research-based advice for community-level practitioners is equally complex: there are no easy answers for questions about how to ensure sustainability of their intervention work. Strategies for increasing the extent of sustainability of the specific interventions included in a research-initiated project (e.g., booster training sessions, targeted technical assistance) may differ substantially from the strategies needed for capacity building to sustain a diversity of health-related activities (e.g., partnership building to provide evidence-based intervention materials across the wide variety of chronic disease interests expressed by community; technical assistance for helping churches build sustainable health ministries and linkages to the healthcare system). Long-term change within community organizations is likely to take a diversity of forms and avenues that reflect the multiple interactions of people, organizations, resources, and interventions.

## Additional files

Additional file 1: 12-month Men’s Participant Survey. This survey was completed by male Project HEAL participants at the 12-month workshop. (PDF 2820 kb)

Additional file 2: 12-month Women’s Participant Survey. This survey was completed by female Project HEAL participants at the 12-month workshop. (PDF 2806 kb)

Additional file 3: 24-month Men’s Participant Survey. This survey was completed by male Project HEAL participants at the 24-month workshop. (PDF 2786 kb)

Additional file 4: 24-month Women’s Participant Survey. This survey was completed by female Project HEAL participants at the 24-month workshop. (PDF 2776 kb)

Additional file 5: 12-month CHA Interview Guide. This CHA interview guide was used by study staff to interview Project HEAL CHAs at the 12-month workshop. (PDF 2662 kb)

Additional file 6: 24-month CHA Interview Guide. This CHA interview guide was used by study staff to interview Project HEAL CHAs at the 24-month workshop. (PDF 2620 kb)

## Abbreviations

CHA: Community health advisor
Community Ministry: Community Ministry of Prince George’s County
HEAL: Health through Early Awareness and Learning
RE-AIM: Reach Effectiveness Adoption Implementation Maintenance
SPSS: Statistical Package for the Social Sciences
