# Supplementary material for: Dimensions of sustainability for a health communication intervention in African American churches: a multi-methods study
Source: Implement Sci. 2017 Mar 28;12:43. doi: 10.1186/s13012-017-0576-x (PMC5371253; doi:10.1186/s13012-017-0576-x)
Supplement: Supplementary file 2 — 12-month Women’s Participant Survey. This survey was completed by female Project HEAL participants at the 12-month workshop. (PDF 2806 kb) [file 13012_2017_576_MOESM2_ESM.pdf]

Project HEAL

Health through Early Awareness and Learning

First Name: ______________________________

Last Name: ______________________________

Address: ______________________________

____

Daytime Phone: ______________________________

Evening Phone: ______________________________

Cell Phone: ______________________________

Cell Carrier/Provider: ______________________________

Email address: ______________________________

Date of birth: ______________________________

In this survey we are going to ask you some questions about cancer and testing for it. You may or may not have any knowledge about these things, and this is ok. Even if you have not heard of cancer or ways of testing for it, this is important for us to know. You can mark a 'not sure' response to any question if you just don't know the answer.

Later during the course of the project we will have one more survey for you to complete on these same topics. We will share our findings back to you so that you know what happened with the project.

We thank you for your patience with this important part of the project.

Please read each question carefully.

Feel free to ask the members of the HEAL staff to assist you if you need any help or have any questions.

Let's begin!

We are interested in your thoughts about the HEAL workshops!

Thank you for taking part in the HEAL project. Your answers on this survey will help to see if the project is making a difference in your community. Please give your honest and best answers.

For each question, please **place an x in the box** that most closely reflects your attitude or feeling. **Other** questions will ask you to **write in** your answer. There are no right or wrong answers - we are interested in your honest opinions.

Please answer the following question.	All	Most	Some	None
How much of the booklets did you read?				

Which workshops did you attend? (mark all that apply)
____ Workshop #1 (Overview of Cancer)
____ Workshop #2 (Breast/Prostate Cancer)
____ Workshop #3 (Colorectal Cancer)

What one thing do you remember most from the workshops you attended? (write in answer)

____

____

Have you shared the knowledge from Project HEAL workshops with any of the below? (Mark all that apply)

____ Family members
____ Friends
____ Co-workers
____ Congregation members
____ None
____ Other: _________________________

The next few questions are about the HEAL Newsletters:

Did you receive newsletters from Project HEAL:

___ Yes
___ No
___ Not sure

How many newsletters do you remember getting?

___ 1
___ 2
___ 3
___ 4 or more
___ Don't remember

How much do you agree or disagree with the following statements?	Strongly Disagree	Disagree	Agree	Strongly Agree	Did not get
I **enjoyed** getting newsletters about the program					
The HEAL newsletters kept me **engaged** in the program.					
The HEAL newsletters kept me **informed** about the program					

What do you like **best** about the newsletters? ____________________________________

What do you like **least** about the newsletters? ____________________________________

The next few questions are about the HEAL text messages:

Did you receive text messages on your phone from the Project HEAL:

___ Yes
___ No
___ Not sure
___ I do not or cannot receive text messages on my phone

How many text messages do you remember getting?

___ 1
___ 2-3
___ 4-6
___ 7 or more
___ Don't remember
___ Does not apply

Do you remember receiving Project HEAL text messages about… (please check **all** that apply)

___ Healthy living
___ Breast cancer
___ Prostate cancer
___ Colorectal cancer
___ Religion/spirituality
___ None of the above
___ Other ________________________________
___ Does not apply

How much do you agree or disagree with the following statements? (please check ONE)	Strongly Disagree	Disagree	Agree	Strongly Agree	Did not get
I **enjoyed** getting text messages about the program					
The HEAL text messages kept me **engaged** in the program.					
The HEAL text messages kept me **informed** about the program					

What do you like **best** about the text messages? _________________________________

What do you like **least** about the text messages? _________________________________

Would you like to continue to receive text messages from Project HEAL?

___ Yes
___ No
___ Not sure

To make sure we have your most current information, please provide your:

Cell Phone Number: ___________________________

Cell Phone Provider: ___________________________

Do you have any **other comments** about the workshop that you have not already mentioned?

____

____

____

____

____

____

About how long has it been since you last visited a doctor for a routine checkup? A routine checkup is a general physical exam, not an exam for a specific injury, illness, or condition.

_____ Within the past year (anytime less than 12 months ago)
_____ Within the past 2 years (1 year but less than 2 years ago)
_____ Within the past 5 years (2 years but less than 5 years ago)
_____ 5 or more years ago
_____ Don't know /Not sure
_____ Never

These next few questions are about colorectal cancer:

Do you agree or disagree with the following statements?	Disagree	Agree	Not Sure
Colorectal cancer is cancer of the colon or rectum.			
Colorectal cancer affects only older White men.			
Risk of colorectal cancer becomes greater as a person gets older.			
Both men and women are at risk for colorectal cancer.			
Colorectal cancer begins as a growth in the colon or rectum.			
Bleeding is a symptom to report to your doctor.			
Colorectal cancer screening is not necessary if there are no symptoms.			
Finding colorectal cancer early will save your life.			
The treatment for colorectal cancer may not be as bad if the cancer is found early.			

<u>We would like to ask you about a test called Fecal Occult Blood test or FOBT:</u>
This test is done to check for colon cancer. It is done at home, using a set of 3 cards, to check if your stools have blood. To do this test, you need to take some of the stool and smear it on the card. Then, you return the card to the doctor's office to be tested.

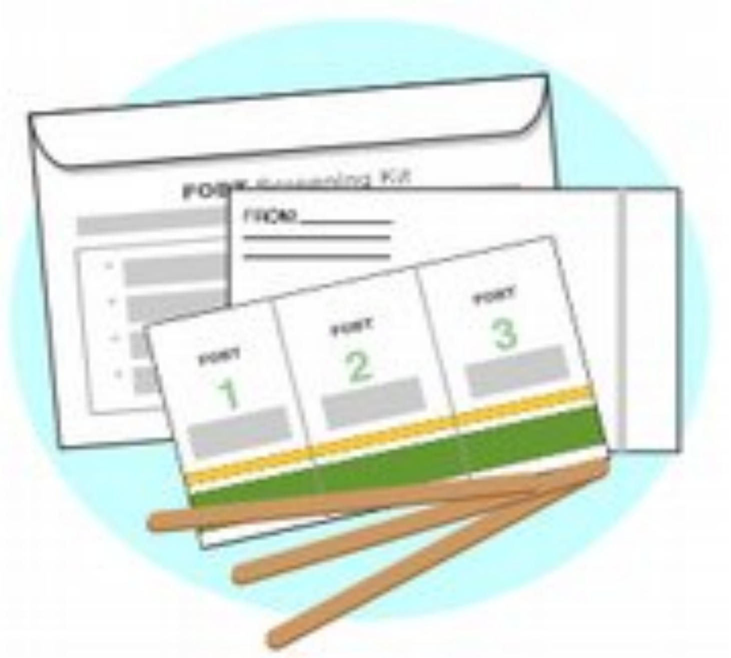

Have you ever <u>heard of</u> this test? **____ Yes ____ No ____ Don't know/Not sure**

If yes, please go to the next page (page 9) of the survey.

If no or don't know/not sure, please skip to page 11 of the survey.

[If you have never heard of a Fecal Occult Blood test (stool test), please SKIP to page 11 of the survey.]

Have you ever used a home kit to do this test? _____ Yes ____ No ____ Don't know/Not Sure

When did you do your last FOBT? ____________ Month _______ Year

How long has it been since you did your last blood stool test using a home kit?

____ In the past year (12 months ago or less)
____ Between 1 and 2 years ago (more than 12 months but less than 24 months ago)
____ More than 2 years ago (More than 24 months ago)
____ Never
____ Don't know /Not sure

Are you thinking about doing a blood stool test in the next 6 months or so? ____ Yes ____ No ____ Don't know

Do you have an appointment to get a blood stool test within the next 6 months? ____ Yes ____ No

Has a health care provider recommended that you do a stool blood test this year, to check your bowel for cancer?

____ Yes ____ No ____ Don't know/Don't remember

	Not at all Confident	Not very Confident	Confident	Very Confident
How confident are you that you can do a home stool blood test?				

Do you agree with the following statements?	Strongly Disagree	Disagree	No Opinion	Agree	Strongly Agree
A FOBT will help find colorectal cancer early.					
A FOBT will decrease your chances of dying from colorectal cancer.					
A FOBT will help you not worry as much about colorectal cancer.					
I am afraid to have a FOBT because I might find out something is wrong.					
A FOBT is embarrassing.					
I do not have time to do a FOBT.					
The cost would keep me from having a FOBT.					
I do not need to do a FOBT because I have no problems.					
I do not have the privacy to do a FOBT.					
I would have trouble having an FOBT because I do not have health insurance.					

<u>We would like to ask you about a test called Sigmoidoscopy:</u>
Sigmoidoscopy is an exam in which a tube is inserted in the rectum to examine the bowel for signs of cancer or other health problems. During the sigmoidoscopy, **<u>you are awake</u>**.

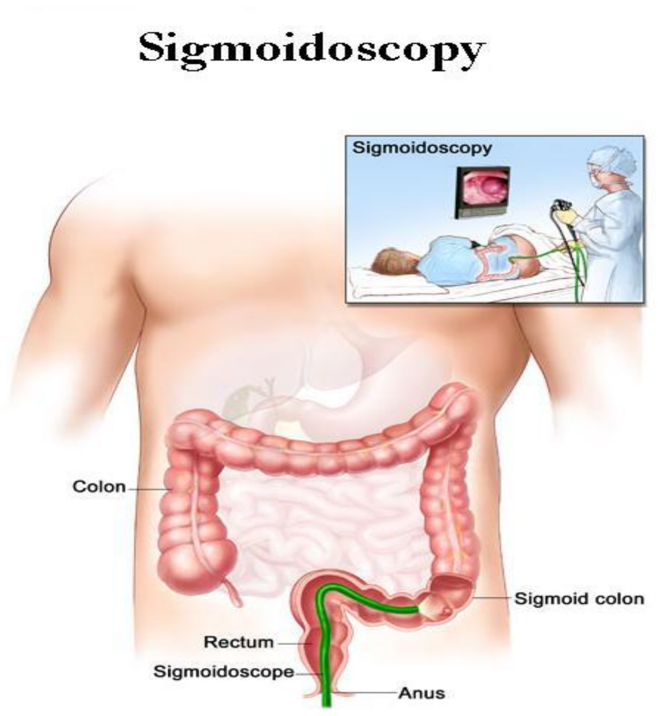

Have you ever <u>heard of</u> a flexible sigmoidoscopy? ____Yes ____ No ____ Don't know/Not sure

If yes, please go to the next page (page 12) of the survey.

If no or don't know/not sure, please skip to page 13 of the survey.

[If you have never heard of a Sigmoidoscopy, please SKIP to page 13 of the survey.]

Have you ever had a flexible sigmoidoscopy? ____ Yes ____ No ____ Don't know/Not sure

If yes, when did you have your last flexible sigmoidoscopy? ________ Month __________ Year

If yes, how long has it been since you had your last sigmoidoscopy?

____ Within the past year (12 months ago or less)
____ Between 1 and 2 years ago (more than 12 months but less than 24 months ago)
____ Between 2 and 3 years ago (more than 24 months but less than 36 months ago)
____ Between 3 and 5 years ago (more than 36 months but less than 60 months ago)
____ Between 5 and 10 years ago
____ More than 10 years ago
____ Never
____ Don't know/Not sure

Are you thinking about having a sigmoidoscopy in the next 6 months or so? ____ Yes ____ No ____ Don't know

Do you have an appointment to get a sigmoidoscopy within the next 6 months? ____ Yes ____ No

Has a health care provider recommended that you have a sigmoidoscopy this year to check your bowel for cancer?

____ Yes ____ No ____ Don't know/don't remember

	Not at all Confident	Not very Confident	Confident	Very Confident
How confident are you that you can schedule and complete a sigmoidoscopy?				

<u>We would like to ask you about a test called Colonoscopy:</u>
A colonoscopy is an exam in which a tube is inserted in the rectum to examine the bowel for signs of cancer or other health problems. During the colonoscopy you are given medicine **<u>to put you to sleep</u>**.

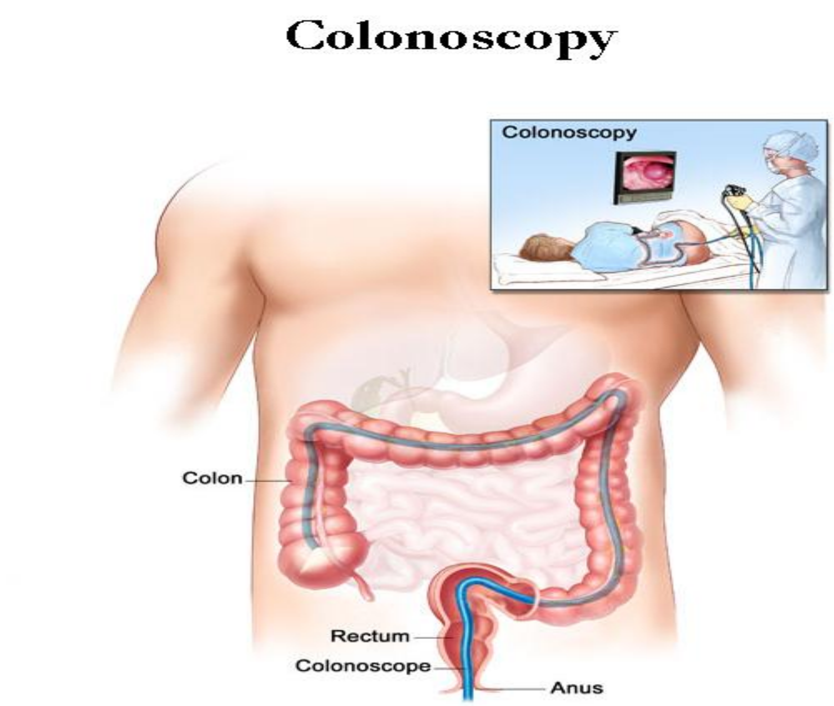

Have you ever <u>heard of</u> a colonoscopy? ____ Yes ____ No ____ Don't know/Not sure

If yes, please go to the next page (page 14) of the survey.

If no or don't know/not sure, please skip to page 16 of the survey.

[If you have never heard of a Colonoscopy, please SKIP to page 16 of the survey.]

Have you ever had a colonoscopy? ____ Yes ____ No ____ Don't know/Not sure

If yes, when did you have your last colonoscopy? ________ Month __________ Year

If yes, how long has it been since you had your last colonoscopy?

____ Within the past year (12 months ago or less)
____ Between 1 and 2 years ago (more than 12 months but less than 24 months ago)
____ Between 2 and 3 years ago (more than 24 months but less than 36 months ago)
____ Between 3 and 5 years ago (more than 36 months but less than 60 months ago)
____ Between 5 and 10 years ago
____ More than 10 years ago
____ Never
____ Don't know/Not sure

Are you thinking about having a colonoscopy in the next 6 months or so? ____ Yes ____ No ____ Don't know

Do you have an appointment to get a colonoscopy within the next 6 months? ____ Yes ____ No

Has a health care provider recommended that you have a colonoscopy this year to check your bowel for cancer?

____ Yes ____ No ____ Don't know/Don't remember

	Not at all Confident	Not very Confident	Confident	Very Confident
How confident are you that you can schedule and complete a colonoscopy?				

Do you agree with the following statements?	Disagree	Agree	Not Sure
A colonoscopy will help find colorectal cancer early.			
A colonoscopy will decrease your chances of dying from colorectal cancer.			
A colonoscopy will help you not worry as much about colorectal cancer.			
I am afraid to have a colonoscopy because I might find out something is wrong.			
A colonoscopy is embarrassing.			
I do not have time to do a colonoscopy.			
The cost would keep me from having a colonoscopy.			
I feel anxious about having a colonoscopy because I don’t really understand what will be done.			
Having a colonoscopy is painful.			
**Do you agree with the following statements?**	**Disagree**	**Agree**	**Not Sure**
Having to follow a special diet and take a laxative or enema would keep me from having a colonoscopy.			
I am afraid to have a colonoscopy because of the possibility there may be bleeding or tearing of the colon.			
Having a colonoscopy might mean that a person is gay or bisexual.			
I would have trouble having a colonoscopy because I do not have health insurance.			

These next few questions are about breast cancer:

A mammogram is an x-ray picture of the breast. When you get a mammogram, your breast is placed between two plastic plates and pressed while the x-ray picture is taken.

When did you have your last mammogram?
___ Within the past 12 months
___ More than 12 months ago
___ I've never had a mammogram

Have you thought about getting a mammogram in the next 6 months?
___ Yes
___ No

Do you have an appointment to get a mammogram within the next 6 months?
___ Yes
___ No

Different women have different ideas about getting a mammogram.
Tell us what you think.

	Yes	No	Not Sure
Are you too busy to get a mammogram?	___	___	___
Would having a mammogram be too painful for you?	___	___	___
Do you have a way to get to the mammogram place?	___	___	___
Can having a mammogram actually cause breast cancer?	___	___	___
Would having a mammogram be too embarrassing for you?	___	___	___
Is the mammogram place open when you have time to go?	___	___	___
Would the cost of a mammogram be a problem for you now?	___	___	___
Would having a mammogram make you worry about having breast cancer?	___	___	___

Tell us what you think a mammogram can do.

	Yes	No	Not Sure
Do mammograms find all breast cancers?	___	___	___
Do you need a mammogram if your breasts feel fine?	___	___	___
Can having a mammogram help set your mind at ease?	___	___	___
Can a mammogram reduce your risk of dying from breast cancer?	___	___	___
Can a mammogram find a breast lump before it's big enough to feel?	___	___	___

Tell us what you think about breast cancer.

	Yes	No	Not Sure
Do most breast lumps turn out to be cancer?	___	___	___
Can bumping or bruising the breasts lead to breast cancer?	___	___	___
Can touching, rubbing, or squeezing the breasts lead to breast cancer?	___	___	___
Are older women more likely to get breast cancer than younger women?	___	___	___
Are Black women more likely to die from breast cancer than White women?	___	___	___
If a breast lump isn't bothering you, is it best just to leave it alone?	___	___	___

Tell us what you think about breast cancer treatment.

	Yes	No	Not Sure
Can breast cancer be treated without removing the breast?	___	___	___
Is the treatment for breast cancer worse than the cancer itself?	___	___	___
Does breast cancer that is found early have a good chance of being cured?	___	___	___

Would you say your chances of getting breast cancer in the next 10 years are:

___ Higher than other Black women your age
___ About the same as other Black women your age
___ Lower than other Black women your age

We want to thank you very much for your participation!
