# Supplementary material for: Dimensions of sustainability for a health communication intervention in African American churches: a multi-methods study
Source: Implement Sci. 2017 Mar 28;12:43. doi: 10.1186/s13012-017-0576-x (PMC5371253; doi:10.1186/s13012-017-0576-x)
Supplement: Supplementary file 3 — 24-month Men’s Participant Survey. This survey was completed by male Project HEAL participants at the 24-month workshop. (PDF 2786 kb) [file 13012_2017_576_MOESM3_ESM.pdf]

 ID# _______________ CH ID # ____________

Project HEAL

Health through Early Awareness and Learning

First Name: ____

Last Name: ____

In this survey we are going to ask you some questions about cancer and testing for it. You may or may not have any knowledge about these things, and this is ok. Even if you have not heard of cancer or ways of testing for it, this is important for us to know. You can mark a 'not sure' response to any question if you just don't know the answer.

This survey is the last survey for Project HEAL. We will share our findings back to you so that you know what happened with the project.

We thank you for your patience with this important part of the project.

Please read each question carefully.

Feel free to ask the members of the HEAL staff to assist you if you need any help or have any questions.

Let’s begin!

Thank you for taking part in the HEAL project. Your answers on this survey will help to see if the project is making a difference in your community. Please give your honest and best answers.

For each question, please **place an x in the box** that most closely reflects your attitude or feeling. **Other** questions will ask you to **write in** your answer. There are no right or wrong answers - we are interested in your honest opinions.

Have you shared the knowledge from Project HEAL workshops with any of the below? (Mark all that apply)

_____ Family members
_____ Friends
_____ Co-workers
_____ Congregation members
_____ None
_____ Other: ______________________________

The next few questions are about the HEAL Newsletters:

Did you receive newsletters from Project HEAL:

___ Yes
___ No
___ Not sure

If no or not sure please <u>skip the next question</u>.

How many newsletters do you remember getting?

___ 1
___ 2
___ 3
___ 4 or more
___ Don’t remember

The next few questions are about the HEAL text messages:

Did you receive text messages on your phone from the Project HEAL:
___ Yes
___ No
___ Not sure
___ I do not or cannot receive text messages on my phone

If no, not sure, or do not/cannot receive, please skip to page 5.

About how often do you remember getting text messages?
___ Less than once per month
___ Once per month
___ Twice per month
___ More than twice per month
___ Don't remember

How much do you agree or disagree with the following statements? (please check ONE)	Strongly Disagree	Disagree	Agree	Strongly Agree	Did not get
I **enjoyed** getting text messages about the program.					
The HEAL text messages kept me **engaged** in the program.					
The HEAL text messages kept me **informed** about the program.					

About how long has it been since you last visited a doctor for a routine checkup? A routine checkup is a general physical exam, not an exam for a specific injury, illness, or condition.

____ Within the past year (anytime less than 12 months ago)
____ Within the past 2 years (1 year but less than 2 years ago)
____ Within the past 5 years (2 years but less than 5 years ago)
____ 5 or more years ago
____ Don't know /Not sure
____ Never

These next few questions are about colorectal cancer:

Do you agree or disagree with the following statements?	Disagree	Agree	Not Sure
Colorectal cancer is cancer of the colon or rectum.			
Colorectal cancer affects only older White men.			
Risk of colorectal cancer becomes greater as a person gets older.			
Both men and women are at risk for colorectal cancer.			
Colorectal cancer begins as a growth in the colon or rectum.			
Bleeding is a symptom to report to your doctor.			
Colorectal cancer screening is not necessary if there are no symptoms.			
Finding colorectal cancer early will save your life.			
The treatment for colorectal cancer may not be as bad if the cancer is found early.			

<u>We would like to ask you about a test called Fecal Occult Blood test or FOBT:</u>
This test is done to check for colon cancer. It is done at home, using a set of 3 cards, to check if your stools have blood. To do this test, you need to take some of the stool and smear it on the card. Then, you return the card to the doctor's office to be tested.

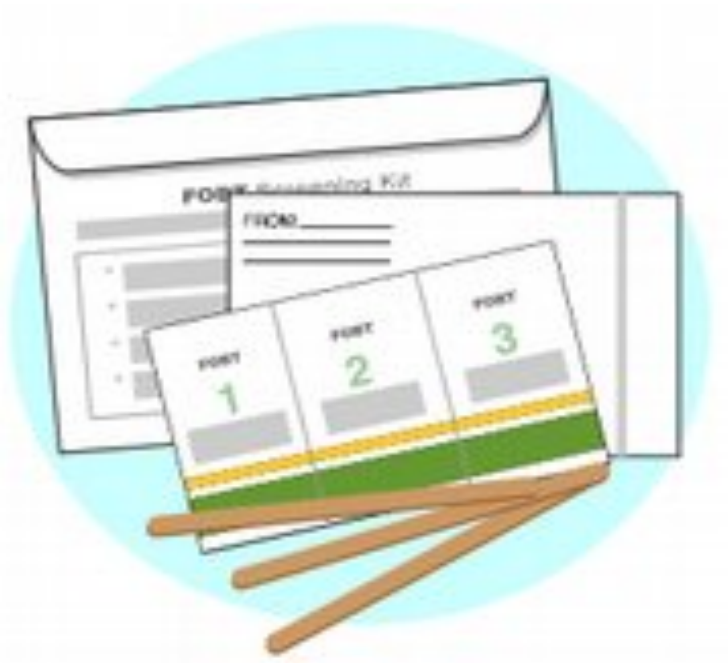

Have you ever <u>heard of</u> this test? **____ Yes ____ No ____ Don't know/Not sure**

If yes, please go to the next page (page 7) of the survey.

If no or don't know/not sure, please skip to page 8 of the survey.

[If you have never heard of a Fecal Occult Blood test (stool test), please SKIP to page 8 of the survey.]

Have you ever used a home kit to do this test? ______ Yes ____ No ____ Don’t know/Not Sure

How long has it been since you did your last blood stool test using a home kit?

____ In the past year (12 months ago or less)
____ Between 1 and 2 years ago (more than 12 months but less than 24 months ago)
____ More than 2 years ago (More than 24 months ago)
____ Never
____ Don’t know /Not sure

Has a health care provider recommended that you do a stool blood test this year, to check your bowel for cancer?

____ Yes ____ No ____ Don’t know/Don’t remember

<u>We would like to ask you about a test called Sigmoidoscopy:</u>
Sigmoidoscopy is an exam in which a tube is inserted in the rectum to examine the bowel for signs of cancer or other health problems. During the sigmoidoscopy, **<u>you are awake</u>**.

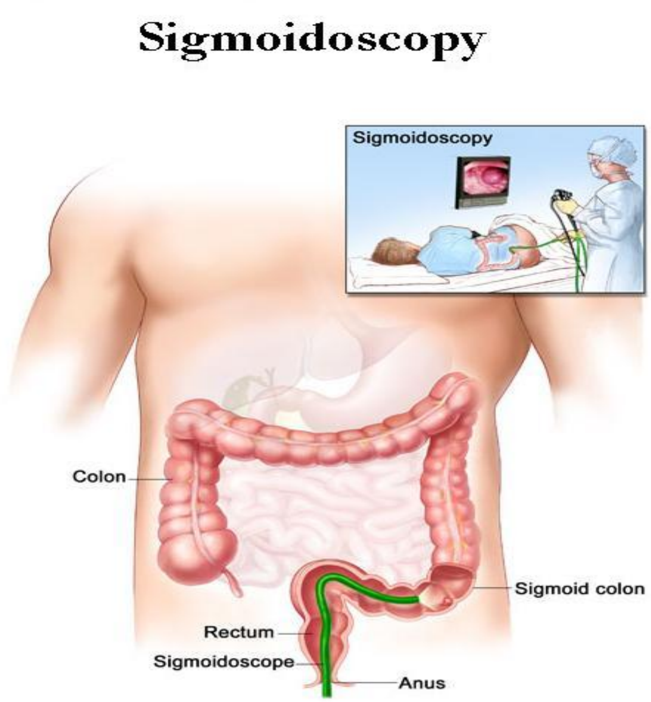

Have you ever <u>heard of</u> a flexible sigmoidoscopy? ____Yes ____ No ____ Don't know/Not sure

If yes, please go to the next page (page 9) of the survey.

If no or don't know/not sure, please skip to page 10 of the survey.

[If you have never heard of a Sigmoidoscopy, please SKIP to page 10 of the survey.]

Have you ever had a flexible sigmoidoscopy? ____ Yes ____ No ____ Don't know/Not sure

If yes, how long has it been since you had your last sigmoidoscopy?

____ Within the past year (12 months ago or less)
____ Between 1 and 2 years ago (more than 12 months but less than 24 months ago)
____ Between 2 and 3 years ago (more than 24 months but less than 36 months ago)
____ Between 3 and 5 years ago (more than 36 months but less than 60 months ago)
____ Between 5 and 10 years ago
____ More than 10 years ago
____ Never
____ Don't know/Not sure

Has a health care provider recommended that you have a sigmoidoscopy this year to check your bowel for cancer?

____ Yes ____ No ____ Don't know/don't remember

<u>We would like to ask you about a test called Colonoscopy:</u>
A colonoscopy is an exam in which a tube is inserted in the rectum to examine the bowel for signs of cancer or other health problems. During the colonoscopy you are given medicine **<u>to put you to sleep</u>**.

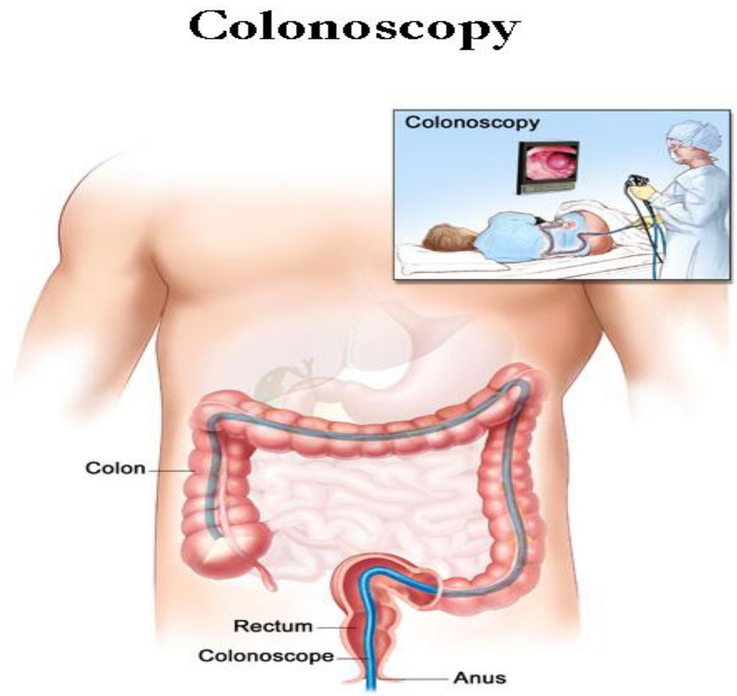

Have you ever <u>heard of</u> a colonoscopy? ____ Yes ____ No ____ Don't know/Not sure

If yes, please go to the next page (page 11) of the survey.

If no or don't know/not sure, please skip to page 13 of the survey.

[If you have never heard of a Colonoscopy, please SKIP to page 13 of the survey.]

Have you ever had a colonoscopy? ____ Yes ____ No ____ Don't know/Not sure

If yes, how long has it been since you had your last colonoscopy?

____ Within the past year (12 months ago or less)
____ Between 1 and 2 years ago (more than 12 months but less than 24 months ago)
____ Between 2 and 3 years ago (more than 24 months but less than 36 months ago)
____ Between 3 and 5 years ago (more than 36 months but less than 60 months ago)
____ Between 5 and 10 years ago
____ More than 10 years ago
____ Never
____ Don't know/Not sure

Has a health care provider recommended that you have a colonoscopy this year to check your bowel for cancer?

____ Yes ____ No ____ Don't know/Don't remember

Do you agree with the following statements?	Disagree	Agree	Not Sure
A colonoscopy will help find colorectal cancer early.			
A colonoscopy will decrease your chances of dying from colorectal cancer.			
A colonoscopy will help you not worry as much about colorectal cancer.			
I am afraid to have a colonoscopy because I might find out something is wrong.			
A colonoscopy is embarrassing.			
I do not have time to do a colonoscopy.			
The cost would keep me from having a colonoscopy.			
I feel anxious about having a colonoscopy because I don't really understand what will be done.			
Having a colonoscopy is painful.			

Do you agree with the following statements?	Disagree	Agree	Not Sure
Having to follow a special diet and take a laxative or enema would keep me from having a colonoscopy.			
I am afraid to have a colonoscopy because of the possibility there may be bleeding or tearing of the colon.			
Having a colonoscopy might mean that a person is gay or bisexual.			
I would have trouble having a colonoscopy because I do not have health insurance.			

These next few questions are about prostate cancer:

Please mark your answers in the boxes to the right.	Yes	No	Not Sure
Is it possible to have prostate cancer even if a man does not have any symptoms?			
Can prostate cancer be treated without removing the prostate itself?			
Are older men more likely to get prostate cancer than younger men?			
Are more African-American men diagnosed with prostate cancer than whites?			
Are African-American men who have fathers or brothers with prostate cancer more likely to get prostate cancer than those who do not?			

Please mark your answers in the boxes to the right.	At this time, doctors are unsure	Definitely Yes	Definitely No	I do not know
Will screening, or testing for prostate cancer, prevent men from dying of prostate cancer?				

Please mark your answers in the boxes to the right.	Yes	No	Not sure
African American men should begin screening for prostate cancer starting at age 40.			
Prostate cancer screening may lead to unneeded biopsies and treatment.			
Not all prostate cancers will kill the man.			
Doctors and experts disagree as to whether prostate cancer screening should be recommended for all men.			

A prostate specific antigen test, or PSA test, is a blood test that measures the level of prostate-specific antigen, a substance produced by the prostate. Increased levels of PSA may be a sign of prostate cancer.

Have you ever had a prostate specific antigen, or PSA test?
___Yes ___ No

[If yes:] Did you have a PSA test: ___ Within the past 12 months
___ More than 12 months ago

A digital rectal examination, or DRE, is an examination in which a doctor inserts a lubricated, gloved finger into the rectum to feel for abnormalities (something that isn't normal).

Have you ever had a digital rectal examination, or DRE test?
__ Yes ___ No

[If yes:] Did you have a DRE test: ___ Within the past 12 months
___ More than 12 months ago

**Please mark your answers in the boxes to the right.**	**Yes**	**No**	**Not Sure**
Having a test for prostate cancer would be too uncomfortable for me, physically.			
I don't have a way to get to the place where they do the test for prostate cancer.			
The place to go for the prostate cancer test isn't open when I have time to go.			

**Did Project HEAL help in your decision making about prostate cancer screening?**	**Not At All**	**A Little Bit**	**Somewhat**	**Quite a Bit**	**A Great Deal**
Help you recognize that a decision needs to be made.					
Prepare you to make a better decision.					
Help you think about the pros and cons of each option.					
Help you think about which pros and cons are most important.					
Help you know that the decision depends on what matters to you most.					
Help you organize your own thoughts about the decision.					
Help you think about how involved you want to be in this decision.					
Help you identify questions you want to ask your doctor.					
Prepare you to talk to your doctor about what matters most to you.					
Prepare you for a follow-up visit with your doctor.					

At this time, would you say you **(choose only one)**:
____ haven't begun to think about making a decision about prostate cancer screening.
____ haven't begun to think about making a decision about prostate cancer screening, but am interested in doing so.
____ are considering the options about prostate cancer screening now.
____ are close to selecting a screening option.
____ have already made a screening decision, but am still willing to reconsider.
____ have already made a screening decision, and am unlikely to change my mind.

Do you have any **other comments** about Project HEAL that you have not already mentioned?

____

We want to thank you very much for your participation!
