# Supplementary material for: Dimensions of sustainability for a health communication intervention in African American churches: a multi-methods study
Source: Implement Sci. 2017 Mar 28;12:43. doi: 10.1186/s13012-017-0576-x (PMC5371253; doi:10.1186/s13012-017-0576-x)
Supplement: Supplementary file 5 — 12-month CHA Interview Guide. This CHA interview guide was used by study staff to interview Project HEAL CHAs at the 12-month workshop. (PDF 2662 kb) [file 13012_2017_576_MOESM5_ESM.pdf]

Project HEAL
Health through Early Awareness and Learning

CHA Interview Guide

Thank you for meeting with me/us today! We are interested in learning about any new activities in your church having to do with health and wellness. Your responses will help inform how we can best partner together on future church-based health activities. There are no right or wrong answers to these questions. Are you ready to begin?

Project HEAL Sustainability

Have there been any more cancer-related workshops conducted at your church since the 3 Project HEAL workshops? ❑ No ❑ Yes ❑ Not Sure

If yes, please let me know the number of workshops and estimated number of attendees for the following topics:

Breast Cancer:	______ number of workshops	_______ number of attendees
Prostate Cancer:	______ number of workshops	_______ number of attendees
Colorectal Cancer:	______ number of workshops	_______ number of attendees
Cancer Overview:	______ number of workshops	_______ number of attendees
Local Resources:	______ number of workshops	_______ number of attendees
Other cancer:	______ number of workshops	_______ number of attendees

Cancer type covered: ____

If yes, who conducted the workshops?

❑ CHA ❑ 2nd CHA
❑ Pastor(s) ❑ Other person(s) (role in the church?) ____________________

If yes, did you use the original HEAL materials, did you modify the HEAL materials, or did you find new materials from somewhere else?

❑ HEAL ❑ Modified HEAL ❑ New materials from somewhere else

If modified or new materials, please describe:

____
____
____

Have there been any other health-related activities sponsored by your church since the 3 Project HEAL workshops (e.g., health fairs or groups)? ❑ No ❑ Yes ❑ Not Sure

If yes, what kind of activities? Answer choices are (*please check all that apply*):

- ❑ Classes/group sessions
- ❑ Brochures distributed (e.g., pamphlets, booklets)
- ❑ Organized health fair(s)
- ❑ Walking or exercise group
- ❑ Health promoting activities/messages in children's programs (such as Sunday school)
- ❑ Testing/Screening
  - If yes, what kind: ____________________________
- ❑ Other: ____
- ❑ Counseling
- ❑ Meetings about health
- ❑ Participated in health fair(s)

If yes, who conducted the activities (*please check all that apply*)?

❑ CHA ❑ 2nd CHA

❑ Pastor(s) ❑ Other person(s) (role in the church?) ____________________

If yes, can you tell me a little more about these activities?

(Interviewer notes/probes: How often? How many participates? Did you partner with other groups?)

____

____

If yes, do you think these would have happened without your participation in Project HEAL? ❑ No ❑ Yes ❑ Not sure

Why or why not?

____

____

What health topics have been covered since the 3 Project HEAL workshops (*Please check all that apply*)

- ❑ Heart disease (including high blood pressure)
- ❑ Stroke
- ❑ Cancer
  - ❑ Breast
  - ❑ Prostate
  - ❑ Colorectal
- ❑ Diabetes
- ❑ Physical Activity
- ❑ Healthy Diet
- ❑ Asthma
- ❑ Aging
- ❑ Obesity/Overweight
- ❑ HIV/AIDS
- ❑ Children's Health
- ❑ Weight Loss
- ❑ Stress Reduction
- ❑ Walking
- ❑ Smoking
- ❑ Other: ____

Has there been a health ministry or health committee that has formed since Project HEAL began? ❑ No ❑ Yes ❑ Not Sure

If yes:

What is the name of the ministry: ______________________

When was your health ministry formed: ______________________

Who leads the health ministry? ______________________

(Interviewer probes: Led by pastor or CHA? Paid or volunteer?)

What is the current meeting schedule?: ______________________

What resources are available to the ministry?: ______________________

If not:

Does your church conduct health activities? ❑ No ❑ Yes ❑ Not Sure

Are you interested in establishing a health ministry? ❑ No ❑ Yes

What future plans do you suggest for your health ministry?

____

____

____

Have there been any additional resources obtained for health activities like space, funds, or equipment? ❑ No ❑ Yes ❑ Not Sure

If yes, what type of resources: ______________________________

Does your church have a formal church wellness policy? ❑ No ❑ Yes ❑ Not Sure

Have there been any changes in church policy that involve health (e.g., healthier meals at church functions, no-smoking zones, etc.?) ❑ No ❑ Yes ❑ Not Sure

If yes, please describe:

____

____

____

Has your church asked for OR received additional technical assistance in health promotion or health education (not including Project HEAL)? ❑ No ❑ Yes ❑ Not Sure

If yes, please describe:

____

____

____

Have you (personally) received further continuing education <u>about cancer</u>? ❑ No ❑ Yes

If yes, please describe:

____

____

____

From where did you learn? ______________________

How long did it take you? ______________________

Have you personally learned or attempted to learn <u>any additional health-related information</u> since your Community Health Advisor training? ❑ No ❑ Yes

If so, on what health topic(s) did you learn more (*Please list all*)?

____

____

____

From where did you learn? ______________________

How long did it take you? ______________________

Were there any outcomes from Project HEAL that were not initially planned (things that happened as a result of Project HEAL)? ❑ No ❑ Yes ❑ Not Sure

If yes, please describe:
____
____
____

Have any other churches, organizations, or individuals contacted your church for information about Project HEAL or other health activities? ❑ No ❑ Yes ❑ Not Sure

If yes, who has contacted your church?:
- ❑ Other churches
  - If yes, number of partners ______
- ❑ Health organizations (e.g., hospitals, health centers, etc.)
  - If yes, number of partners ______
- ❑ Universities or colleges
  - If yes, number of partners _______
- ❑ Other: ____

To your knowledge, has your church participated in any other research projects since the Project HEAL workshops: ❑ No ❑ Yes ❑ Not Sure

(If yes, describe the project(s) and your partner(s)) [Does NOT include Project HEAL]
____
____
____

Pastor's Participation in Project HEAL

What actions did the pastor or church leadership take in promoting the HEAL workshops? Answer choices are: (*please check all that apply*)

- ❑ Mentioned workshops in sermon
- ❑ Announced workshops during announcements
- ❑ Included workshop announcements in church bulletins
- ❑ Invited members to attend
- ❑ Attended the workshops
- ❑ None
- ❑ Other: (please describe)

____

How many workshops did Pastor attend? ❑ 0 ❑ 1 ❑ 2 ❑ 3 ❑ Cannot recall

How would you rate Pastor's level of enthusiasm about the Project HEAL workshops?

1 2 3 4 5

Not enthusiastic Very enthusiastic

How long has the lead pastor been in place in your church? ___________

Other Clergy or Lay Health Ministry Leader Participation in Project HEAL

Were there other clergy or lay health ministry leaders (other than self/other CHA) involved in Project HEAL from your church? ❑ No ❑ Yes

If yes, what actions did the other clergy or lay health ministry leader take in promoting the HEAL workshops? Answer choices are: (*please check all that apply*)

- ❑ Mentioned workshops in sermon
- ❑ Announced workshops during announcements
- ❑ Invited members to attend
- ❑ Announced workshops during ministry meetings
- ❑ Attended the workshops
- ❑ None
- ❑ Other: (please describe)

____

How many workshops did other clergy or lay health ministry leaders attend?

❑ 0 ❑ 1 ❑ 2 ❑ 3 ❑ Cannot recall

If other clergy or lay health ministry leaders attended, how enthusiastic were they about the Project HEAL workshop?

1	2	3	4	5
Not enthusiastic				Very enthusiastic

Community Health Advisor Insights

In your opinion, what was the most positive aspect of your volunteer work with Project HEAL?

___

___

___

Was there anything about your work with Project HEAL that you did not like, or that did not work well?

___

___

___

Looking back, would you have done anything differently about your volunteer work with Project HEAL?

___

___

___

Would you like to add any more comments on your experience as a Community Health Advisor with Project HEAL?

___

___

___

Thank you for your time and for participating in Project HEAL.
