# Supplementary material for: Dimensions of sustainability for a health communication intervention in African American churches: a multi-methods study
Source: Implement Sci. 2017 Mar 28;12:43. doi: 10.1186/s13012-017-0576-x (PMC5371253; doi:10.1186/s13012-017-0576-x)
Supplement: Supplementary file 6 — 24-month CHA Interview Guide. This CHA interview guide was used by study staff to interview Project HEAL CHAs at the 24-month workshop. (PDF 2620 kb) [file 13012_2017_576_MOESM6_ESM.pdf]

Church ____________________ ZB CHA Exit Survey 24mo

Project HEAL

Health through Early Awareness and Learning

*Please tell us about any new health/wellness activities in your church **in the last 12 months**.*

1. Have there been any more cancer-related workshops conducted at your church ***in the last 12 months***? ❑ No ❑ Yes ❑ Not Sure

   If yes, cancer type covered:____

2. Have there been any other health-related activities sponsored by your church ***in the last 12 months*** (e.g., health fairs or groups)? ❑ No ❑ Yes ❑ Not Sure

   If yes, what kind of activities? Answer choices are (*please check all that apply*):

   ❑ Classes/group sessions
   ❑ Brochures distributed (e.g., pamphlets, booklets)
   ❑ Organized health fair(s)
   ❑ Walking or exercise group
   ❑ Health promoting activities/messages in children's programs (such as Sunday school)
   ❑ Testing/Screening
   ❑ Other: ____
   ❑ Counseling
   ❑ Meetings about health
   ❑ Participated in health fair(s)

3. What health topics have been covered ***in the last 12 months*** (*Please check all that apply*)

   ❑ Heart disease (including high blood pressure)
   ❑ Stroke
   ❑ Cancer
     ❑ Breast
     ❑ Prostate
     ❑ Colorectal
   ❑ Diabetes
   ❑ Physical Activity
   ❑ Healthy Diet
   ❑ Other: ____
   ❑ Asthma
   ❑ Aging
   ❑ Obesity/Overweight
   ❑ HIV/AIDS
   ❑ Children's Health
     ❑ Weight Loss
   ❑ Stress Reduction
   ❑ Walking
   ❑ Smoking

4. Has there been a health ministry or health committee that has formed ***in the last 12 months***? ❑ No ❑ Yes ❑ Not Sure

5. Has your church put in place a formal church wellness policy ***in the last 12 months***?

   ❑ No ❑ Yes ❑ Not Sure

6. Would you like to add any more comments on your experience as a Community Health Advisor with Project HEAL? (Use the back of this sheet for more space)

____
